# Severe Encephalatrophy and Related Disorders From Long-Term Ketamine Abuse: A Case Report and Literature Review

**DOI:** 10.3389/fpsyt.2021.707326

**Published:** 2021-10-01

**Authors:** Linying Liu, Haijian Huang, Yongbin Li, Ruochen Zhang, Yongbao Wei, Weiwei Wu

**Affiliations:** ^1^Fujian Medical University Cancer Hospital, Fujian Cancer Hospital, Fuzhou, China; ^2^Shengli Clinical Medical College of Fujian Medical University, Fuzhou, China; ^3^Department of Pathology, Fujian Provincial Hospital, Fuzhou, China; ^4^Department of Urology, Fujian Jianou Hospital, Jianou, China; ^5^Department of Urology, Fujian Provincial Hospital, Fuzhou, China; ^6^Department of Neurology, Union Hospital, Fujian Medical University, Fuzhou, China

**Keywords:** ketamine, abuse, encephalatrophy, disorder, mental activity

## Abstract

Ketamine is a glutamate N-methyl D-aspartate receptor antagonist and an anaesthetic agent that has been effectively used to treat depression. However, ketamine has also been increasingly used for recreational purposes. The dissociative side-effects of ketamine use, such as hallucinations, are the reason for abuse. Additionally, long-term ketamine abuse has been highly associated with liver-gallbladder and urinary symptoms. The present study reports the case of a **28-year-old young male adult** with an 8-year history of daily inhalation of ketamine. We investigated the association between ketamine abuse and the mechanism of its adverse effects, particularly encephalatrophy, and attempted to find a link between these disorders. These results would help us to better understand ketamine usage, ketamine abuse effects and the addictive mechanism. To the best of our knowledge, the present case is the first report of severe brain atrophy related to ketamine abuse. Details of the patient are presented and the mechanism of the encephalatropy-associated ketamine abuse is discussed. Furthermore, organ dysfunction following chronic ketamine abuse may indicate that the side effects are the result of comprehensive action on multiple regions in the brain.

## Introduction

Ketamine is a derivative of phencyclidine that was first successfully synthesised by the American pharmacist Calvin Stevens in 1962. Ketamine was initially used as an effective veterinary anaesthetic. Then, it became commercially available for human use as a rapid-acting intravenous anaesthetic. However, the adverse side-effects were quickly realised and its use in treatment was reduced. Great importance has been attached to ketamine again with the increased number of patients with a depressive disorder and with the rapid-acting (within hours) and sustained (lasting up to 7 days) antidepressant effects of ketamine in traditional anti-depressant drug-resistant patients (1, 2), and it has been demonstrated that such effects may be associated with an opioid effect (3). Additionally, experiments have demonstrated that ketamine use can reduce suicidal ideation rapidly (4–6), while that effects on suicidal ideation are partially independent of its effects on mood (7). The therapeutic action on both depression and suicidal behaviour may be highly significantly correlated to some microRNAs (8).

Patients who abuse ketamine develop non-specific clinical symptoms (9), particularly neuropsychiatric symptoms. Liver-gallbladder symptoms, such as cholestasis, biliary dilatation or abnormal liver function, and urinary symptoms, such as increased frequency, hydronephrosis, chronic kidney injury and renal failure have also been reported (10–15). Ketamine may also cause sclerosing cholangitis in patients with COVID-19 (16). In addition, Cheng-Chung Liu has reported pulseless ventricular tachycardia associated with ketamine abuse (17). In the present case report, a patient with an 8-year history of daily inhalation of ketamine developed brain atrophy. Although ketamine addicts with neuropsychiatric symptoms have been described, such a patient with severe encephalatrophy has not been reported. To the best of our knowledge, the present case is the first report of severe encephalatrophy related to ketamine abuse. Written informed consent was obtained from the family of the patient.

## Case Description

**Figure 1:**
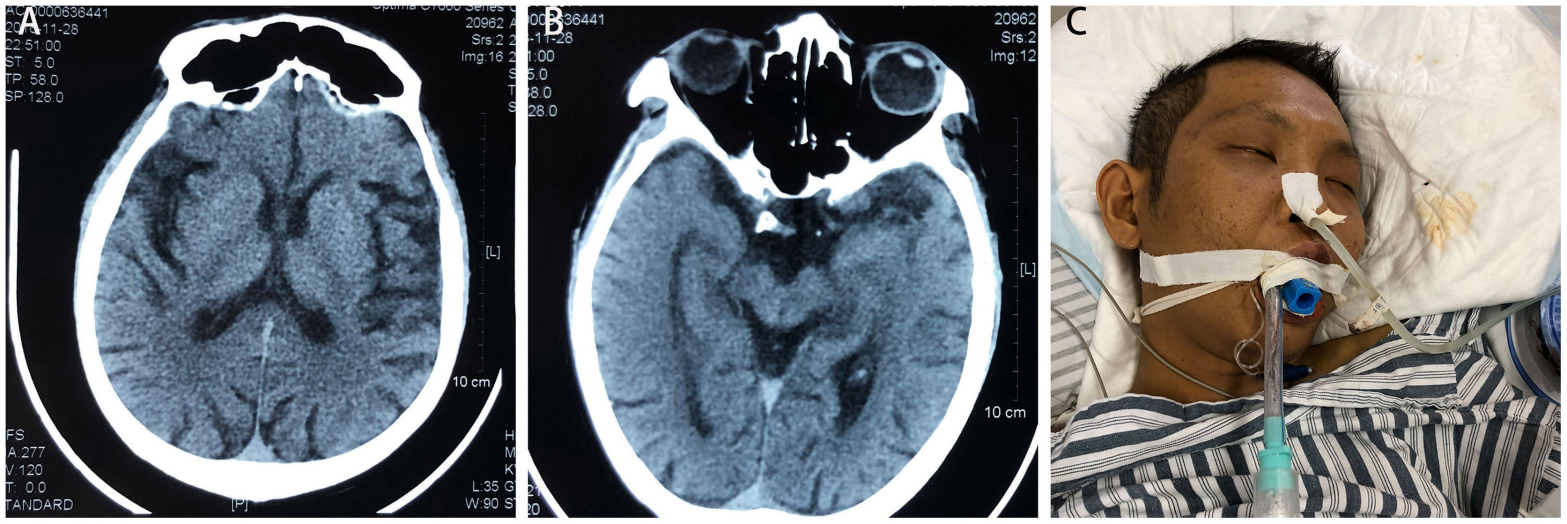
Craniocerebral computed tomography scan indicates wide and deep cerebral sulcus, flattened cerebral palsy, expanded ventricles, thickened cerebellum and reduced brain volume **(A,B)**. **(C)** The patient was placed on a ventilator but soon died of persistent epilepsy and multiple organ failure.

A 28-year-old male was admitted to the respiratory department of Jian'ou Hospital (Fujian, China) with repeated coughing and yellow phlegm in 2018. Family members complained that the patient had a history of ketamine abuse of about five to seven times monthly for 8 years. The last ketamine inhalation was the day before he was admitted to the hospital, **according to a family member**. During the past 5 years, he had been experiencing weakness of the lower limbs, numbness, sensory disorder, decreased muscle strength, difficulty climbing stairs, recurrent frequent urination and urgency and pain during urination. These symptoms persisted and were not significantly relieved by several conservative treatments but were worsened by repeated convulsions, aching in the hepatic region and jaundice 2 years ago. Then, he was diagnosed with “autoimmune hepatitis, chronic cholecystitis and cholangitis.” **At that time**, a craniocerebral computed tomography (CT) scan revealed a widened and deepened cerebral sulcus, the cerebral palsy became flattened, the ventricles expanded, the cerebellum was thickened, and brain volume decreased (Figure 1). Electromyography revealed a central motor and sensory conduction disorder, and the motor nerve conduction velocity of bilateral tibial nerves was slower, indicating ketamine-related brain atrophy. His liver function normalised completely after dexamethasone treatment **(15 mg for treatment and 5 mg for maintenance for more than 3 months). The patient planned to have cholecystectomy surgery 1 year later, but the operation was cancelled due to abnormal liver function and the development of renal failure**. A CT examination revealed a constricted bladder with dilatation of the bilateral upper urinary tract, moderate hydronephrosis and medullary sponge kidney with multiple renal papillary calcifications. Subsequently, the patient's urine volume decreased gradually until he was not producing urine. He underwent haemodialysis treatment twice per week. After a sudden fall about 2 months before this hospitalisation, the patient was unable to walk and was confined to a wheelchair. He could not speak but made some slurred sounds, and he was conscious 2 weeks before admission.

**The patient and his family members denied a history of cerebral disease or alcohol abuse**. He was somnolent and jaundiced at admission. A physical examination revealed that muscle strength of both lower limbs was 0 and that of the upper limbs was 3. Routine blood testing revealed an increase in the number of leukocytes and a decrease in the number of erythrocytes. The haemoglobin value was 65 g/l. A faecal occult blood test was positive at 3+. His liver function tests (LFTs) were abnormal (serum bilirubin 283.3 mol/L, alkaline phosphatase 1,804 IU/L, alanine transaminase 19 IU/L, yGT 762 IU/L, and albumin 28.2 g/dL). Other blood tests revealed renal failure (urea 26.97 mmol/L and creatinine 375 μmol) and myocardial failure (NT-pro BNP >35,000 ng/L). Cerebrospinal fluid (CSF) was tested three times, but the routine test, culture test, and syphilis test were all negative. A chest and abdominal CT scan demonstrated pneumonia and bilateral kidney abnormalities. The patient's condition deteriorated rapidly with regular pneumonia, blood transfusions and haemodialysis treatments. During treatment, he presented with sweating, clammy limbs and repeated convulsions. Then, he required intensive care support. Symptoms improved initially with symptomatic treatment, but worsened quickly, accompanied by dyspnoea and purple lips. After convulsing again, the patient slipped into a deep coma and was intubated and maintained on a ventilator. In the end, he died from brain damage and dysfunction with other multiple organ dysfunctions.

## Discussion

Ketamine is a non-competitive N-methyl D-aspartate (NMDA) receptor antagonist commonly used initially as a human intravenous anaesthetic. However, its use has decreased due to side effects, particularly addiction. Ketamine addiction is related to the interference of glutamate neurotransmission, which influences the establishment of the subcortical regions, such as nucleus accumbens, which is associated with addictive behaviours (18). Studies also show that there are specific antidepressant properties of glutamatergic medications targeting NMDA receptors (19, 20) and effective reduction of suicidal ideation (5). Furthermore, a small dose of ketamine can relieve pain in advanced tumour patients (14, 21, 22), but side effects must be considered. The side-effects of ketamine abuse or misuse (23), except addiction, include neurotoxicity, cognitive dysfunction, adverse events associated with mental status, psychotomimetic effects, uropathy effects, liver-gallbladder effects and cardiovascular events. **Research has shown that chronic ketamine use may lead to brain atrophy, particularly during the time of brain development** **(****24**, **25****), and the anti-depressive effects may be associated with increases in brain area volume** **(****20****)**. **But to our knowledge, we are the first to report a young adult suffering from severe brain atrophy after long-term abuse of ketamine**.

**The side effects of ketamine abuse or misuse are commonly seen. In our case, the patient was diagnosed with autoimmune hepatitis, chronic cholecystitis, cholangitis and renal failure**. It is unknown why he first presented with neurological symptoms from the weakness of the lower limbs, numbness, sensory disorder and decreased muscle strength. He then repeatedly convulsed, suddenly lost the ability to speak and died of brain dysfunction. Symptoms, such as weakness of the lower limbs and aphasia (26, 27), indicate brain damage or brain functioning zone damage, but his brain atrophy may not be the same as common degeneration (28) which is associated with ageing. No signs of intracranial infection or any other brain disease were detected on CSF tests, such as cerebral infarction from the CT scans, except atrophy. However, a patient with status epilepticus reportedly also developed brain atrophy and some similar neurological symptoms after using ketamine (29). Therefore, it is likely that brain atrophy is a side-effect of chronic ketamine use. However, because ketamine is a dissociative drug, it is trivial for physicians to determine that a patient may appear with neuropsychiatric symptoms while overlooking a brain image to check whether any degenerative brain changes are occurring at the same time. **This could be due to the few reports of brain atrophy associated with ketamine usage** **(****24**, **25****)**.

There are several subtypes of encephalatrophy also known as brain atrophy, and it can be diagnosed by CT scan (30). As people age, it is common to see brain atrophy, and in recent years, many studies have demonstrated that brain atrophy is associated with dementia or Alzheimer's disease (31). However, most surveys have focused on the relationship between brain atrophy and cognitive dysfunction and have found that brain atrophy leads to cognitive dysfunction (32–34). **No study has investigated the sensory or motor symptoms related to brain atrophy**. As our patient was very young and his symptoms were unique, such as loss of speaking ability and repeated convulsions, we infer that the brain atrophy in a ketamine addict may not be the same as common degeneration, as the patient may also have damage to specific brain functioning zones. **Additionally, a reduction in frontal grey matter volume can be detected by MRI among long-term ketamine users** **(****35****); therefore, it was reasonable for us to find brain atrophy on CT in our case**.

Other lower affinity pharmacological targets of ketamine include, but are not limited to, γ-aminobutyric acid (GABA), dopamine, serotonin, sigma, opioids and cholinergic receptors, as well as voltage-gated sodium and hyperpolarisation-activated cyclic nucleotide-gated channels (1, 9). These may lead to diverse effects with chronic ketamine use. For example, GABA is the principal inhibitory neurotransmitter in the adult brain, and the balance between inhibitory and excitatory synaptic transmission is essential for normal neuronal communication and brain function. Several neurodevelopmental diseases have been associated with the GABAergic system during brain development (36) by impairing the integration of GABAergic neurons in the cerebral cortex. However, the impact on the adult brain is unknown. **In our case, the patient presented with convulsions, which may have been the result of inadequate inhibitory effects, such as GABA, but** the functional relevance of the actions of ketamine on GABA_A_ receptors is unclear (9). **A recent study** **(****37****)** **reported that a single subanaesthetic dose of ketamine for a rapid and sustained anti-depressive effect is the result of the action of GluN2B-NMDARs on GABA interneurons, indicating that ketamine may not act directly on other receptors (except NMDA receptors) but includes indirect effects. Additionally, the abusive dose of the drug is similar to that for treatment purposes or even higher** [doses used for recreational ketamine use may range between 1 and 2 mg/kg (i.v.), 50 and 150 mg (i.m.), 100 and 500 mg (oral) or 30 and 400 mg (intranasal insufflation) (38, 39)], **which increases the addiction risk. However, the dose-effects on ketamine receptors are notable**.

It is well-established that ketamine abuse can be neurotoxic, resulting in cognitive impairment and psychotic states (40). Additionally, it was found to be associated with certain receptors. A related study (41) suggested that ketamine neurotoxicity of the brain may occur due to activation of apoptotic pathways in the prefrontal cortex. Another hypothesis (42) of ketamine neurotoxicity is associated with AMPA (α-amino-3-hydroxy-5-methyl-4-isoxazole-propionic acid) receptors. Furthermore, a survey revealed that changes in membrane AMPA receptors and synaptic function induced by ketamine are mediated by abnormal phosphorylation of the tau protein at specific sites (43). The mechanism of ketamine neurotoxicity remains unclear. A case reported that a woman with major depressive disorder used long-term increasing doses of ketamine from 50 mg/week to 2 g/d for an anti-depression response (44); the woman eventually became a ketamine addict accompanied by loss of consciousness, dissociative immobility and amnesia. This indicates that ketamine is a double-edged sword and that neurotoxicity may be associated with dose. More treatment-related receptors work at low doses, whereas overdoses cause activities of some receptors, such as AMPA, to be modified or damaged, leading to adverse events related to neurotoxicity. As our patient slipped into a deep coma, we confirmed that he was neurotoxic. It is unknown whether the encephalatrophy of our patient was related to ketamine neurotoxicity. More studies on the association between different receptors and doses are needed.

**An association has been found between addictive drugs and brain volumetric changes. One case reported that the recreational use of N**_**2**_**O for 5 months led to encephalatrophy and cognitive dysfunction** **(****45****), and the patient's symptoms including encephalatrophy improved after treatment. Furthermore, brain volumetric changes are also observed among other addicts, besides those abusing ketamine** **(****46****), such as methamphetamine abusers** **(****47****)** **[and methamphetamine at abstinence** **(****48****)] and alcoholics** **(****49**, **50****)** **[and alcohol abstinence** **(****51****)]. The mechanisms are unknown but may be associated with microRNA** **(****52****), glucose metabolism** **(****53****)** **or some brain receptors** **(****54****)**. However, the brain damage would improve after abstinence. In our case, infectious factors were excluded due to the three negative CSF results. His encephalography was associated with ketamine use, as he was a young man without a cerebral disease history. Ketamine has a direct negative inotropic effect and an indirect stimulatory effect due to activation of the sympathetic system by blocking nicotinic acetylcholine receptors located on cerebral perivascular sympathetic nerves, resulting in diminished neurogenic vasodilation and normal blood flow to the brainstem (55), which may explain the patient's encephalatrophy. The patient was anaemic, which was discovered after the brain atrophy, but the anaemia could not explain such severe encephalatrophy.

Our patient also developed renal failure and some other diseases, which may have been related to his brain atrophy. It has been reported that levels of methylguanidine and guanidinosuccinate increase in uraemic patients, which is related to symptoms of convulsions and brain atrophy (56, 57). The patient's renal function tests showed that creatinine and blood urea nitrogen were not significantly elevated with regular haemodialysis treatment, **and brain atrophy was observed before renal failure. Thus, we excluded the possibility of renal-mediated brain atrophy**. The LFTs normalised after the administration of **dexamethasone**. What's more, with a negative liver-related **auto-antibody test on the last admission**, we inferred that **the autoimmune hepatitis was treated properly. However, the patient also presented with abnormal liver function during the last admission, which may have been a result of ketamine or chronic cholecystitis and cholangitis. Ketamine causes liver damage** **(****13**, **58**, **59****). As we are unsure when the abnormal live function and brain atrophy began, the brain atrophy may have been liver mediated. However, as the liver symptoms lasted a short time and were treated quickly, it is unlikely that such severe brain atrophy was caused by abnormal liver function**. We infer that the severity of the brain atrophy may not be related to abnormal renal and liver function. **Additionally, the patient did not have a long history of using some drugs except dexamethasone more than 3 months. Dexamethasone may lead to reduced brain size** **(****60****). However, dexamethasone was used after the patient was found to have severe brain atrophy. Thus, we excluded other drug-mediated brain atrophy**.

It is difficult to confirm the mechanism of these non-specific symptoms, and the factors associated with the severity of brain dysfunction. Several hypotheses for the potential relationship between ketamine abuse and urinary tract or liver-gallbladder damage have been proposed. Among these, most theories involve ketamine acting through receptors on the liver-gallbladder or urinary tract, as ketamine is metabolised in the liver by microsomal cytochrome enzymes and the metabolites are excreted into the urine and bile. An animal study (61) showed that injecting NMDA into the dorsal motor nucleus of the vagus increases gallbladder motility and its effect can be abolished by administering ketamine, which explains the relevant gallbladder symptoms. Therefore, it is plausible that the various symptoms of different organs reflect brain damage through organ axes of the central nervous system. That scenario corresponds with our patient's present brain damage followed by dysfunction of other organs.

In conclusion, this is the first report of long-term ketamine use associated with severe encephalatrophy and other neurological symptoms. The mechanism of ketamine side- effects may be the result of comprehensive action of multiple receptors associated with the dose, and the various symptoms may result from organ axes of the central nervous system by some receptors and related miRNA changes. We suggest that brain atrophy be regarded as a common side-effect and that a brain examination should be performed on ketamine addicts. We could observe early receptor-relevant protein changes from visual cerebral volumes changes to have a better understanding of the mechanism of action of ketamine, its side effects, or even prevent the adverse events from progressing. More studies are needed on the ketamine mechanism between receptors and molecules.

## Data Availability Statement

The original contributions presented in the study are included in the article/supplementary material, further inquiries can be directed to the corresponding authors.

## Ethics Statement

The studies involving human participants were reviewed and approved by Ethics Committee of Fujian Provincial Hospital. The patients/participants provided their written informed consent to participate in this study. Ethical review and approval was not required for the animal study because no animal included in the study. Written informed consent was obtained from the individual(s) for the publication of any potentially identifiable images or data included in this article.

## Author Contributions

LL wrote the first draught of the manuscript. YL and RZ treated the patient. HH created the image and collected the clinical information. WW and YW edited the paper and sponsored the study. All authors contributed to the article and approved the submitted version.

## Funding

This study was supported by Startup Fund for scientific research, Fujian Medical University (2019QH1050) and high-level hospital foster grants from Fujian Provincial Hospital, Fujian Province, China (2020HSJJ18).

## Conflict of Interest

The authors declare that the research was conducted in the absence of any commercial or financial relationships that could be construed as a potential conflict of interest.

## Publisher's Note

All claims expressed in this article are solely those of the authors and do not necessarily represent those of their affiliated organizations, or those of the publisher, the editors and the reviewers. Any product that may be evaluated in this article, or claim that may be made by its manufacturer, is not guaranteed or endorsed by the publisher.
